# Rational design of Raman-labeled nanoparticles for a dual-modality, light scattering immunoassay on a polystyrene substrate

**DOI:** 10.1186/s13036-015-0023-y

**Published:** 2016-01-07

**Authors:** Nathan D. Israelsen, Donald Wooley, Cynthia Hanson, Elizabeth Vargis

**Affiliations:** Department of Biological Engineering, Utah State University, 4105 Old Main Hill, Logan, UT 84322 USA

**Keywords:** High-throughput, Immunoassay, Multiplexing, Nanoparticle, Raman Spectroscopy, Surface-Enhanced Raman Spectroscopy/Scattering (SERS)

## Abstract

**Background:**

Surface-enhanced Raman scattering (SERS) is a powerful light scattering technique that can be used for sensitive immunoassay development and cell labeling. A major obstacle to using SERS is the complexity of fabricating SERS probes since they require nanoscale characterization and optical uniformity. The light scattering response of SERS probes may also be modulated by the substrate used for SERS analysis. A typical SERS substrate such as quartz can be expensive. Polystyrene is a cheaper substrate option but can decrease the SERS response due to interfering Raman emission peaks and high background fluorescence. The goal of this research is to develop an optimized process for fabricating Raman-labeled nanoparticles for a SERS-based immunoassay on a polystyrene substrate.

**Results:**

We have developed a method for fabricating SERS nanoparticle probes for use in a light scattering immunoassay on a polystyrene substrate. The light scattering profile of both spherical gold nanoparticle and gold nanorod SERS probes were characterized using Raman spectroscopy and optical absorbance spectroscopy. The effects of substrate interference and autofluorescence were reduced by selecting a Raman reporter with a strong light scattering response in a spectral region where interfering substrate emission peaks are minimized. Both spherical gold nanoparticles and gold nanorods SERS probes used in the immunoassay were detected at labeling concentrations in the low pM range. This analytical sensitivity falls within the typical dynamic range for direct labeling of cell-surface biomarkers using SERS probes.

**Conclusion:**

SERS nanoparticle probes were fabricated to produce a strong light scattering signal despite substrate interference. The optical extinction and inelastic light scattering of these probes was detected by optical absorbance spectroscopy and Raman spectroscopy, respectively. This immunoassay demonstrates the feasibility of analyzing strongly enhanced Raman signals on polystyrene, which is an inexpensive yet non-ideal Raman substrate. The assay sensitivity, which is in the low pM range, suggests that these SERS probe particles could be used for Raman labeling of cell or tissue samples in a polystyrene tissue culture plate. With continued development, this approach could be used for direct labeling of multiple cell surface biomarkers on strongly interfering substrate platforms.

**Electronic supplementary material:**

The online version of this article (doi:10.1186/s13036-015-0023-y) contains supplementary material, which is available to authorized users.

## Background

**Fig. 1:**
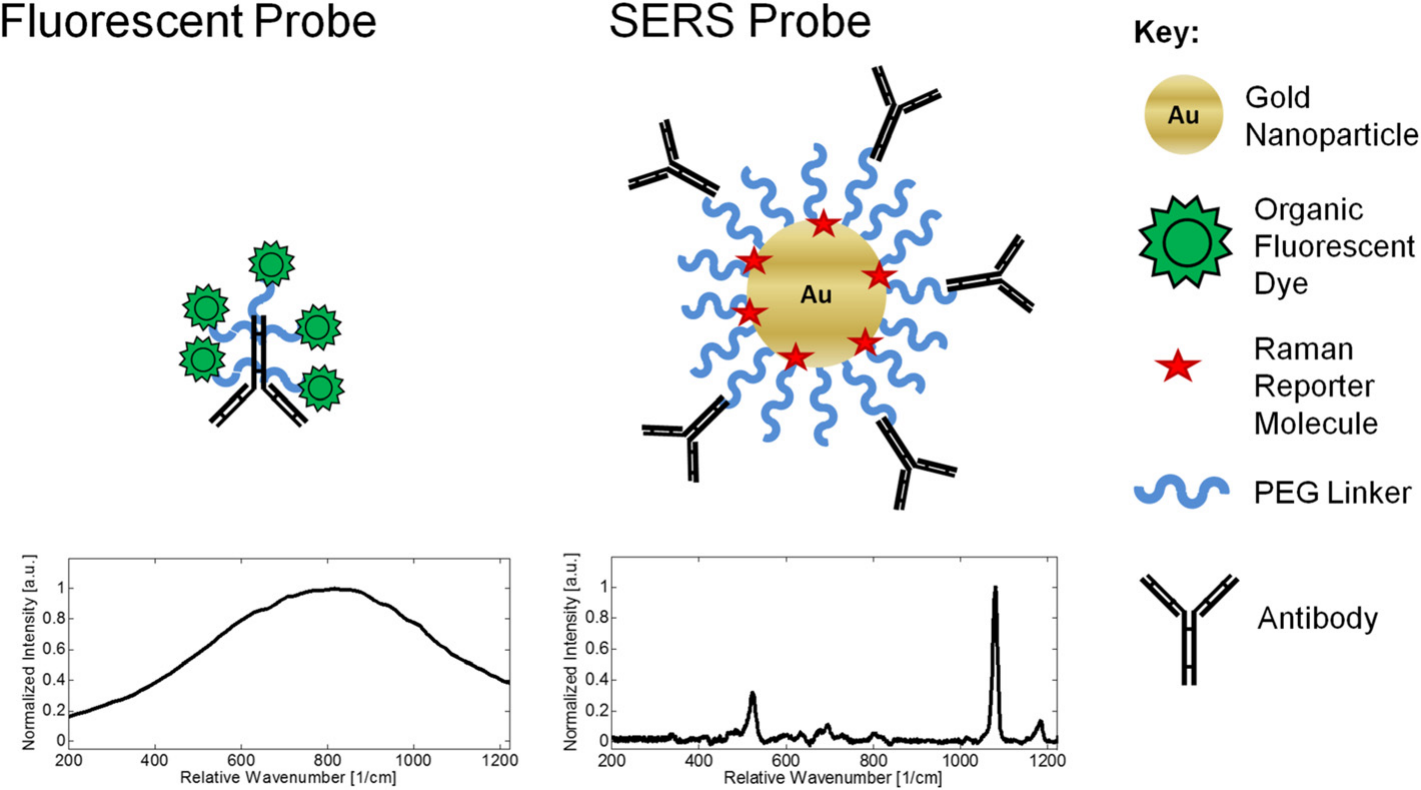
A comparison of the spectral emission width and structure of fluorescent and SERS probes. The spectral emission width of SERS probe is much narrower than fluorescent probes. The narrow spectral emission width of SERS probes enables the light scattering signal from multiple SERS probes to be detected simultaneously without peak overlap

Surface-enhanced Raman spectroscopy (SERS) has the potential to address the clinical need to develop direct cell labeling platforms with both high-throughput and multiplexing capabilities. One example of this need is the classification of hematological malignancies, which require increased multiplexing capacity, as there are 60 recognized subtypes with unique biomarker profiles, pathological characteristics, and required treatments [1–8]. Commonly used immunoassay detection methods such as fluorescence can only detect 3–5 unique analytes, due to wide fluorescent spectral emission peaks, as illustrated in Fig. 1 [9, 10]. This limitation has prompted interest in developing nanoparticle-based optical probes for multiplex biomarker analysis.

Several different types of nanoparticles have been used for multiplex immunoassays including colorimetric nanoparticles [11, 12], quantum dots [13, 14], and SERS probes [15–18]. Of these, SERS probes have the narrowest emission peaks and the greatest multiplexing capacity [10, 19]. It is estimated that SERS probes could label up to 100 different biomarkers with little spectral overlap [10, 19]. The development of a SERS immunoassay using polystyrene microplates for protein binding has the potential to meet current clinical needs for high-throughput detection of multiple analytes.

In addition to their multiplexing capacity, SERS probes have several other characteristics that make them useful in immunoassay development. SERS is a light scattering technique with an emission lifetime of approximately 10^−14^ seconds, which is shorter than a typical fluorescence emission lifetime of approximately 10^−8^ seconds [20]. The short emission lifetime of SERS decreases the amount of time that the reporter molecule remains in an excited state and reduces photobleaching [21]. Another advantage of SERS probes is their ability to withstand extreme environmental conditions (changes in humidity, pH, and ionic strength) while maintaining a strong emission signal [22, 23]. Since SERS probes are resistant to environmental changes, they have been used for *in vivo* detection of biomarkers [24, 25]. Finally, because of the light scattering nature of SERS probes, a single light source can be used to excite multiple SERS probes at the same time [23, 26]. Each of these characteristics makes SERS probes ideal for robust and sensitive multiplex immunoassays.

SERS probes are fabricated using gold or silver nanoparticles labeled with Raman reporter molecules and antibodies for detection and targeting. The metallic nanoparticle core enhances the electromagnetic field near the Raman reporter molecules, resulting in an average light scattering enhancement of 10^4^ - 10^6^ times [27]. This enhanced electromagnetic field is caused by excitation of the nanoparticle’s surface electrons and is referred to as localized surface plasmon resonance (LSPR). LSPR is an electron-wave resonance state caused by the oscillation of the nanoparticles’ electrons in response to incident light. LSPR produces intense nanoparticle absorption and scattering at a specific wavelength referred to as the LSPR peak wavelength. The LSPR wavelength is influenced by the size, shape, and dielectric properties of the nanoparticles [28]. Nanoparticle geometry and LSPR characteristics can be tuned to match a Raman system’s excitation wavelength, resulting in increased light scattering enhancement and greater SERS probe assay sensitivity [29, 30].

SERS probes used in direct cell labeling applications can typically be detected in the low pM to low nM nanoparticle range [31, 32]. Table 1 lists references, published from 2013 to 2015, showing the dynamic range for SERS probes used in direct cell labeling applications [33–37]. Assay sensitivity and dynamic range vary widely and depend on multiple factors including nanoparticle geometry, Raman reporter density, and Raman system throughput [29, 38]. Another factor that directly influences SERS assay sensitivity is the substrate used for sample analysis. Commonly used substrates for SERS-based immunoassays include gold or silver coated surfaces [15–17, 39, 40], quartz microscope slides [41, 42], or mica sheets [43]. Less expensive polystyrene substrates are traditionally not used in SERS analysis due to their strong light scattering background signal and intrinsic fluorescence [44]. The goals of this research are to develop a SERS assay on a polystyrene substrate and to determine the effect of substrate inference on the assays sensitivity and dynamic range.

**Table 1:** SERS assay dynamic range

Nanoparticle Type	Raman Reporter	Dynamic Range	Lower Limit of Detection (LLOD)	Polystyrene Substrate?	Reference
Gold Nanorods (LSPR 815nm)	NIR Dyes	1 - 100 pM	1 pM	No	[33]
Silver Nanospheres Dimers	4-MBA	1 -500 pM	1 pM	No	[34]
Silica Coated Gold Nanospheres	S420, S421, S440	1-400 pM	1 pM	No	[35]
Silica Coated Gold Nanospheres	S420,S440	1-1200 pM	1 pM	No	[36]
Silica Coated Gold Nanospheres	S420, S421, S440	0.8 - 800 pM	0.8 pM	No	[37]
60 nm Gold Nanospheres	DTTC Iodide	76 - 500 pM	76 pM	Yes	**
Gold Nanorods (LSPR 779nm)	DTTC Iodide	45 - 500 pM	45 pM	Yes	**

The typical dynamic range for SERS-based immunoassays and cell labeling ranges from 1–1200 pM SERS probe [33–37]. The dynamic range is expressed as the actual number density of the SERS particles and should not be confused with the concentration of Raman reporter molecule added to the particles surface. Raman reporter concentration will typically range from 1,200 - 10,000 reporter molecules per colloid [82]. Note: ** SERS probe detection limits and Raman-based assay development methods presented in this paper

**Fig. 2:**
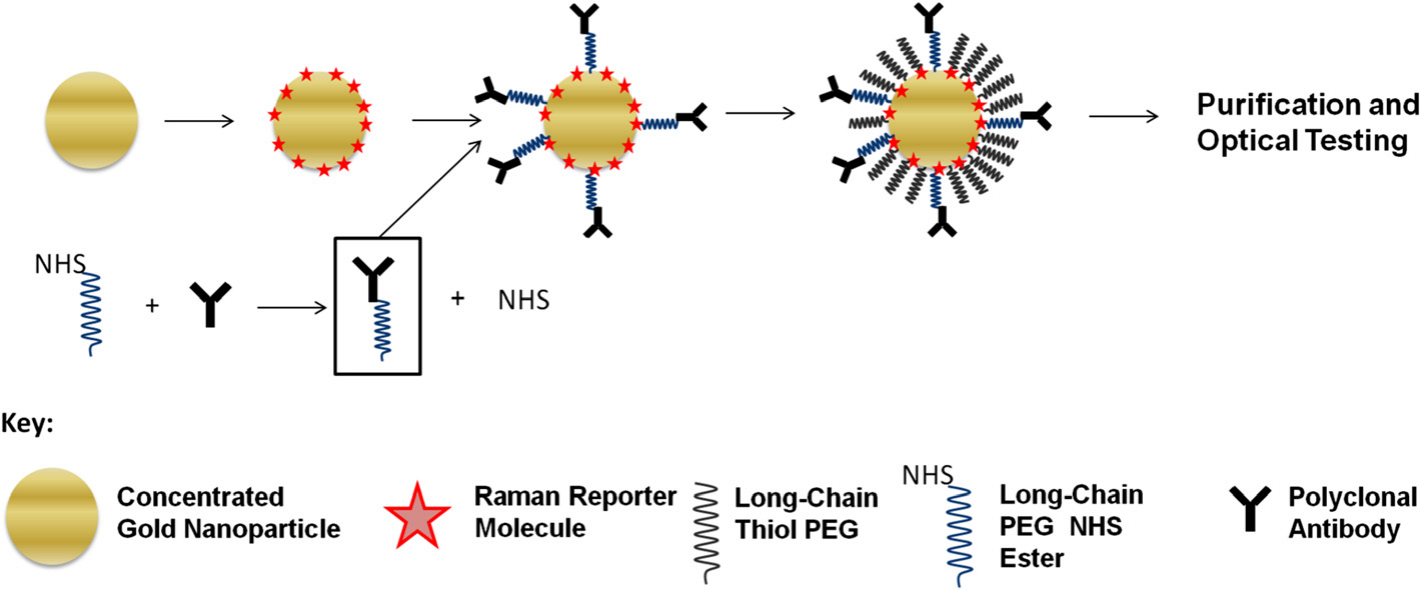
Synthesis schema for the production of SERS probes. The labeling and functionalization of concentrated gold nanoparticles during the fabrication of SERS probes is a multistep process, which can be optimized for use with strongly interfering substrates such as polystyrene

In this study, we developed Raman-labeled nanoparticles for an immunoassay using a polystyrene microplate substrate. SERS probes used in the assay were synthesized as shown in Fig. 2. First, gold nanoparticles were labeled with Raman reporter molecules. After Raman reporter binding, polyclonal antibodies were covalently modified with a long-chain polyethylene glycol (PEG) linker molecule and then conjugated to the probe surface. The particles were further stabilized with additional PEG and then purified by centrifugation. This approach was used to produce SERS probes for a light scattering immunoassay. Optical absorbance spectroscopy was used to quantify elastic scattering from the gold nanoparticles, while Raman spectroscopy was used to detect inelastic light (Raman) scattering. This dual-modality approach can be used to characterize SERS probes prior to cell or tissue labeling studies and to assess the influence that substrate interference may have on the labeling application.

## Results and Discussion

### Raman reporter selection and optimization of SERS probe optical response on a polystyrene substrate

**Fig. 3:**
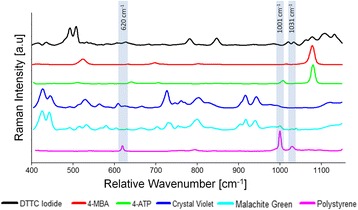
Raman reporter selection and polystyrene peak overlap. To avoid substrate interference, Raman reporters were selected that had very little peak overlap with the Raman spectrum of polystyrene. Major peaks observed in the polystyrene spectra are found at 620 cm^−1^, 1002cm^−1^, and 1032 cm^−1^

To determine the optimal Raman reporter molecule for strong light scattering enhancement on a polystyrene substrate, several commonly used Raman reporter molecules were surveyed using a custom Raman microscope system designed in-house (See Additional file 1: Figure S1). These Raman reporters included 4-aminothiolphenol (4-ATP), 4-mercaptobenzoic acid (4-MBA), crystal violet, malachite green, and 3,3′- diethylthiatricarbocyanine (DTTC) iodide. Each Raman reporter was mixed with a nanoparticle solution and analyzed both on a non-interfering quartz substrate and on a highly-interfering polystyrene substrate. The response of each Raman reporter was evaluated to determine the required acquisition time, the intensity of major Raman peaks, and the position of those peaks relative to the Raman peaks of polystyrene. It was determined that each of the reporter molecules was a viable candidate for use on a polystyrene substrate because the major peaks observed in the polystyrene spectrum (620 cm^−1^, 1002 cm^−1^, and 1032 cm^−1^) did not overlap major peaks seen in each Raman reporter spectra (Fig. 3).

**Fig. 4:**
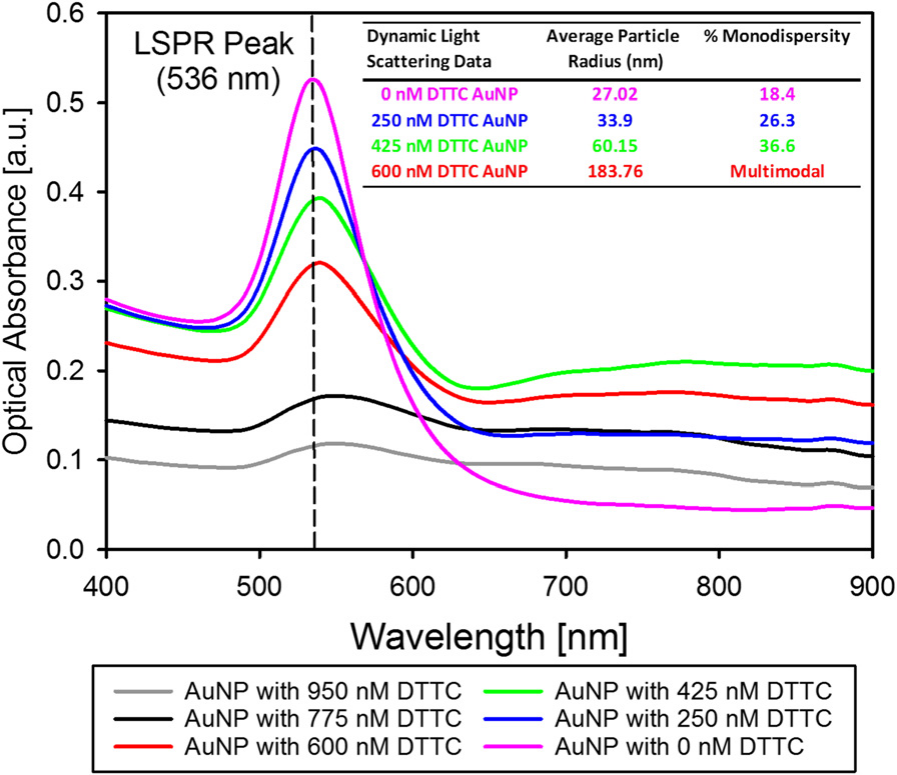
Raman reporter-induced aggregation. When high concentrations of Raman reporter were used during the reporter labeling process, nanoparticle aggregation occurred. The aggregation process results in a LSPR shift and a reduction in LSPR intensity, which can reduce SERS enhancement. DTTC iodide-induced aggregation was observed by monitoring the LSPR peak spectrum of the nanoparticles and by using dynamic light scattering

After the Raman reporter was added to the nanoparticle solutions, a significant increase in nanoparticle aggregation was often observed. This aggregation was caused by ion-induced displacement of the citrate-capping layer on the nanoparticle surface [45]. The formation of nanoparticle aggregates was monitored by measuring the shift in the LSPR wavelength after reporter addition and by dynamic light scattering (Fig. 4). Depending on the mean size of nanoparticle aggregates and the extent of the LSPR shift, the aggregation process can produce significant changes in the particles’ SERS response. In some cases, Raman reporter-induced aggregation results in an increase in the SERS response due to the formation of intense nanoparticles hot spots [46]. Without using methods for precise control of hot spot formation and aggregation, this effect may also result in inconsistent Raman enhancement and decreased assay reproducibility [47]. During fabrication of a typical batch, Raman reporter-encoded nanoparticles had limited, and highly controlled, aggregation as seen in Fig. 4, which shows the LSPR shift and mean particles size of gold nanoparticles that were labeled with DTTC iodide.

To reduce nanoparticle aggregation, the concentration of the Raman reporter molecule was maintained at less than 600 nM during the labeling process. In addition to reducing nanoparticle aggregation, this low reporter concentration was required to promote uninhibited antibody binding. Ionic reporter molecules, such as DTTC iodide, crystal violet, and malachite green, produced nanoparticle aggregation when used at high concentrations. On the other hand, thiol-containing molecules such as 4-ATP and 4-MBA successfully stabilized the particle surface, resulting in very little nanoparticle aggregation. Based on these initial observations, reporter molecules such as 4-ATP or 4-MBA seem to be ideally suited for maintaining a monodispersed solution of Raman encoded nanoparticles. We observed that thiol-containing Raman reporters are excellent at stabilizing the particle surface, but they resulted in poor SERS enhancement with our custom Raman system (Additional file 2: Figure S2). In fact, it has been reported that 4-MBA may result in poor SERS enhancement unless added to the nanoparticle surface at concentrations high enough to form a self-assembled monolayer [48][49].

Because DTTC iodide provided the best optical response on our custom Raman microscope system, it was selected as the Raman reporter for SERS labeling (Additional file 2: Figure S2). DTTC iodide is a near-infrared dye with a maximum absorption at 765nm. Among the Raman reporter molecules surveyed, DTTC iodide was unique because its maximum absorption point is near our Raman system’s excitation wavelength (785nm). Matching the Raman system’s excitation wavelength to the molecule’s absorption band further amplified inelastic light scattering through resonance Raman enhancement [29, 50, 51]. This resonance contribution resulted in an increase in the Raman signal and a decrease in substrate interference. The non-overlapping profile of DTTC iodide-labeled SERS probes proved to be the best solution to maximize SERS probe enhancement on a polystyrene substrate.

### Antibody conjugation and particle functionalization

**Fig. 5:**
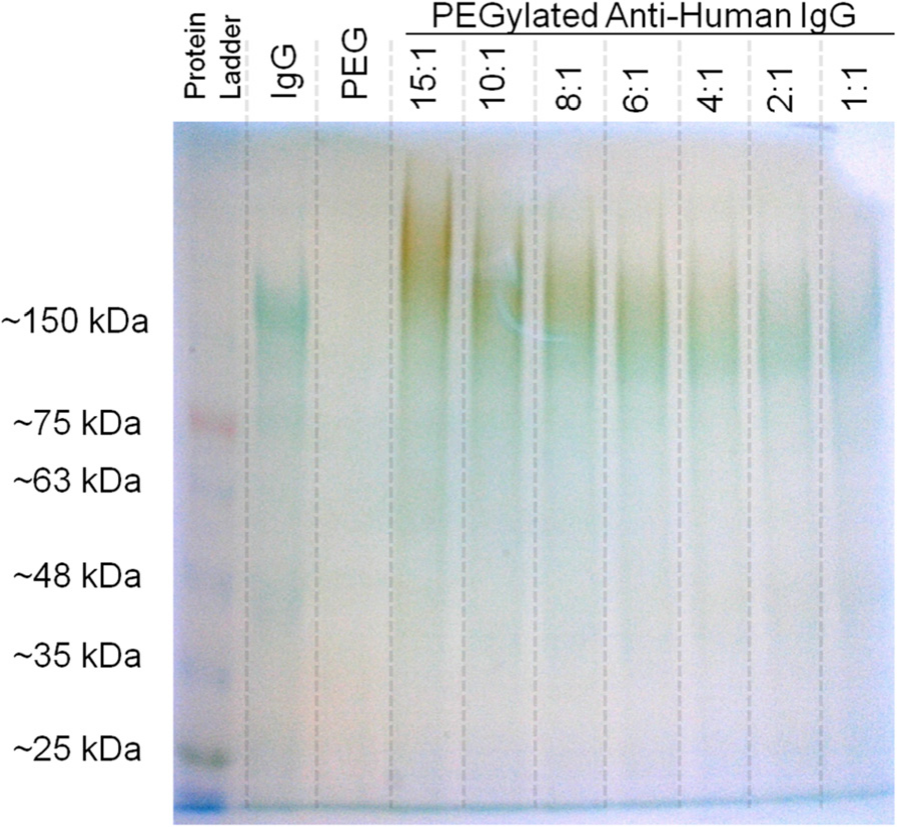
SDS-PAGE analysis of PEGylated antibodies. SDS-PAGE analysis confirmed successful antibody PEGylation. The gel was stained using Coomassie Blue to detect protein (blue/green on the gel) and a barium chloride iodine mixture to detect PEG (brown). The image contrast for Fig. 5 was uniformly adjusted to highlight each individual band in the gel

Anti-human IgG antibodies were bound to the gold nanoparticles’ surface using an activated orthopyridyldisulfide (OPSS)-PEG-NHS molecule that covalently binds to primary amine groups on the protein. When the activated PEG molecule is resuspended in a protein solution, two competing reactions will occur: conjugation and hydrolysis (Additional file 3: Figure S3). Reaction conditions were modified to promote conjugation of the activated PEG molecule. The conjugation reaction was performed with the following PEG:antibody molar conjugation ratios: 1:1, 2:1, 4:1, 6:1, 8:1, 10:1, and 15:1. Successful conjugation of OPSS-PEG to the antibodies was verified using sodium dodecyl sulfate - polyacrylamide gel electrophoresis (SDS-PAGE) analysis. As shown in Fig. 5, the molecular weight of the PEGylated proteins increases proportionally to the increasing PEG:protein ratio as indicated by the position of the PEGylated proteins with relation to the protein ladder. Also, PEG staining of the gel using a 5 % barium chloride solution followed by an iodine/iodide solution showed that the amount of bound PEG increased consistently with the increasing PEG:protein ratio. The results from the protein gel confirm that successful conjugation occurred with addition of activated OPSS-PEG to the protein solution.

PEGylated antibodies were bound to the nanoparticle surface through gold-disulfide binding. The disulfide group on the OPSS-PEG molecules has been used frequently for gold nanoparticle functionalization and binds to the solid gold surface through a semi-covalent gold-sulfur bond [52–55]. An advantage of using activated OPSS-PEG for protein binding is that it avoids potential problems with protein disulfide reduction caused by the use of thiol-based agents in gold nanoparticle functionalization [56]. The binding of OPSS-PEG to the nanoparticles’ surface was confirmed by the increased particle stability caused by the addition of OPSS-PEG to the nanoparticles solution. OPSS-PEG stabilized gold nanoparticles showed increased stability and aggregation resistance even in solutions of high ionic strength. Increasing the stability of the OPSS-PEG stabilized particles was an important step for antibody functionalization and SERS-probe targeting.

### Addressing the challenge of particle stability

One challenge of developing antibody-conjugated SERS probes is balancing nanoparticle stability and protein stability. To maintain nanoparticle stability, ultra-pure water (18.2 MΩ-cm) is commonly used for nanoparticle dilution and resuspension [57]. On the other hand, protein solubility and proper protein function require the use of buffers such as phosphate-buffered saline (PBS) or tris-buffered saline (TBS). These buffers maintain the hydration layer around the protein, which results in a stable protein conformation and solubility [58–60]. In this study, the key to maintaining the balance between protein and nanoparticle stability was adding PEGylated antibodies at an optimized antibody to gold nanoparticle ratio of 200:1. It was observed that if PEGylated antibodies were added to the gold nanoparticle solution at a ratio significantly higher than this, ion-induced particle aggregation would occur. Alternatively, if PEGylated antibodies were added at a lower ratio, the nanoparticles would remain stable but protein aggregation and insolubility would occur.

After the addition of PEGylated antibodies, the nanoparticles were further stabilized with a layer of thiol-polyethylene glycol (SH-PEG). SH-PEG provides steric stabilization, which makes the nanoparticles resistant to the effect of ion-induced aggregation. SH-PEG functionalized SERS probe nanoparticles were fabricated, providing a consistent optical response for several weeks. These particles could also be centrifuged and dispersed without signal loss or particle aggregation (Additional File 4: Figure S4). During the fabrication process, the aggregation state was monitored using optical absorbance spectroscopy and dynamic light scattering to ensure that surface functionalization did not disrupt particle stability.

### Development of a SERS immunoassay using spherical gold nanoparticles and gold nanorods

**Fig. 6:**
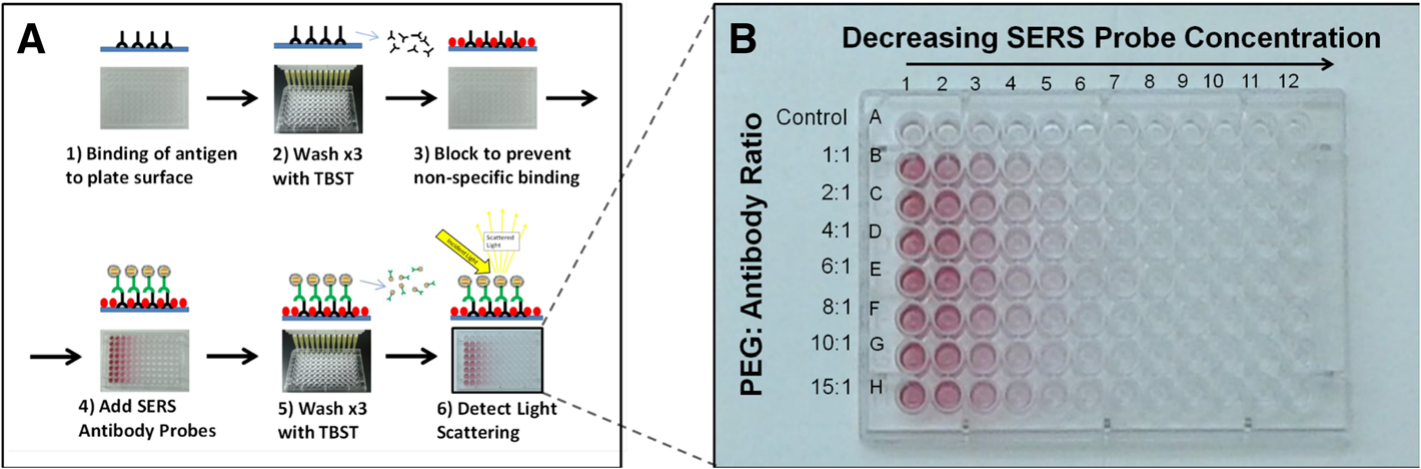
SERS immunoassay development. Fig. 6a presents a visual protocol for the development of a light scattering immunoassay. Step 1: Antigen is non-covalently bound to the polystyrene plate. Step 2: Buffer is used to remove unbound antigen. Step 3: The polystyrene surface is blocked to prevent non-specific binding. Step 4: SERS probe detection antibody is added to the plate. Step 5: Wash buffer is used to remove unbound SERS probe. Step 6: Light scattering from the SERS probe is measured for assay quantification. The resulting assay shows specific binding of the SERS probe to polystyrene embedded human IgG (Fig. 6b)

Antibody-conjugated SERS probes were developed and tested in a light scattering immunoassay format. A visual protocol for the light scattering assay is presented in Fig. 6a. PEG:protein ratios of 1:1, 2:1, 4:1, 6:1, 8:1, 10:1, 15:1 were evaluated to determine the effect of PEG conjugation on the probes light scattering response. The resulting immunoassay shows specific binding of the SERS probe nanoparticles to human isotype IgG, bound to the polystyrene plate (Fig. 6b). The unconjugated SERS probe control condition (Fig. 6b, row A) shows very little specific binding. In addition, PEG conjugation ratios of up to 15:1 have a minimal effect on the ability of the SERS probes to bind to the immunoassay plate.

To quantify SERS probe binding, two batches of DTTC SERS probes were fabricated. One batch was developed using spherical gold nanoparticles (AuNP) and the second was fabricated with gold nanorods (AuNR). A consistent Raman reporter-labeling ratio was used for both AuNP and AuNR particles so that the signal response could be compared based on different nanoparticle geometries rather than on the concentration of Raman reporter. The optical density of the Raman-labeled nanoparticles was determined to be 20.3 ± 3.3 OD_(536nm)_ and 20.1 ± 1.1 OD_(779nm)_ for AuNP and AuNR, respectively. After fabrication and purification, the LSPR wavelengths and Raman intensities of the two SERS probe geometries were compared in a light scattering immunoassay.

### Quantification of spherical gold nanoparticles and gold nanorods using optical absorbance spectroscopy

**Fig. 7:**
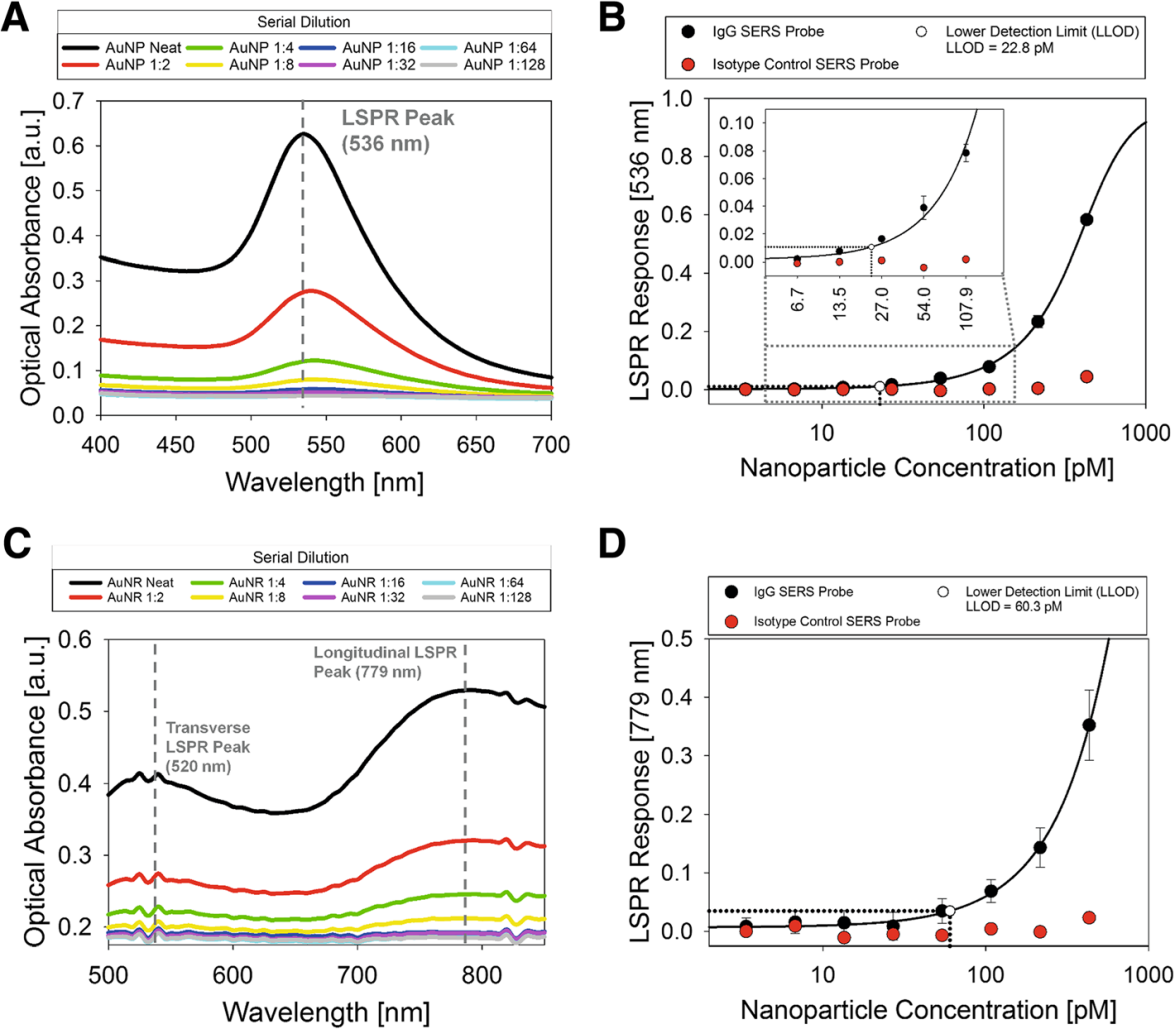
AuNP and AuNR light scattering immunoassay with detection by optical absorbance spectroscopy. After antibody-conjugated gold nanoparticles were bound to the immunoassay plate, the assay response could be detected using optical absorbance spectroscopy. The absorbance spectrum of each well was recorded and the intensity of the major LSPR peaks for both AuNP and AuNR SERS probes was determined (Fig. 7a, c). The LSPR response was correlated to the nanoparticle concentration and a standard curve and sensitivity plot was developed (Fig. 7b, 7d). The curve was fit using five-parameter logistic equation. LLOD values for AuNP and AuNR assays detected using optical absorbance spectroscopy were 22.8 pM and 60.3 pM, respectively

The assay was quantified using optical absorbance spectroscopy. In a typical assay, SERS probes were diluted across 12 wells of the immunoassay plate using a 1:2 serial dilution, so that the full assay range from the nM to low pM range could be assayed in a single plate. To quantify the assay response, the absorbance of each well was measured over a range of 400–850 nm and the wavelength and intensity of the LSPR peak was determined. The AuNP SERS probes had a single LSPR peak at 536nm while the AuNR SERS probes had both a transverse LSPR peak at 520nm and a longitudinal LSPR peak at 779 nm (See Fig. 7a and c). The maximum LSPR peak intensity was then plotted against the concentration of nanoparticle added to each well (Fig. 7b and d). The resulting data points were fit with a five-parameter logistic curve. The assay's sensitivity limits were calculated according to the Clinical and Laboratory Standard Institutes EP17-A2 guidelines [61, 62]. The lower limit of detection (LLOD) for the assay is defined as the lowest concentration that could be distinguished from the blank with 95 % confidence [63]. Based on this calculation, the LLOD for the AuNP and AuNR assays were determined to be 22.8 pM and 60.3 pM respectively.

### Quantification of spherical gold nanoparticles and gold nanorods using SERS

**Fig. 8:**
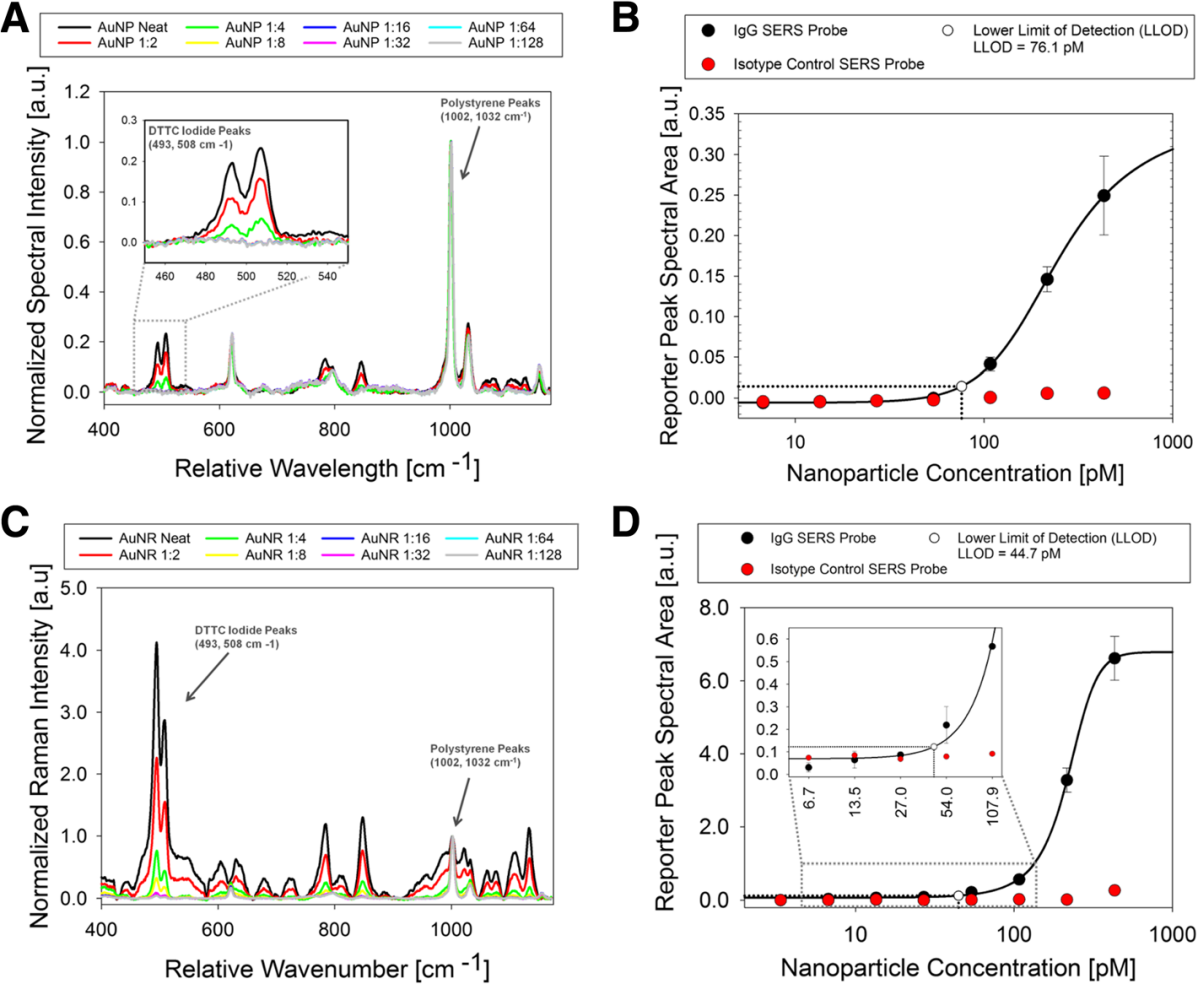
AuNP and AuNR light scattering immunoassay with detection by SERS. Using the 785nm custom Raman microscope, the Raman spectra of the AuNP and AuNR SERS probes bound to the polystyrene plate were recorded. Fig. 8a shows the Raman spectrum of DTTC iodide labeled AuNP SERS probes. The spectrum shows characteristic peaks corresponding to DTTC iodide at 493 cm^−1^ and 508 cm^−1^ and to polystyrene at 1002 cm^−1^ and 1032 cm^−1^. By solving for the area under each reporter peak, an AuNP SERS probe standard curve was developed (Fig. 8b). The curve was fit using a five-parameter logistic equation and the LLOD was calculated as 76.1 pM. The Raman spectra of AuNR SERS probes were also acquired (Fig. 8c). A standard curve and sensitivity plot shows that the LLOD for the AuNR SERS probe assay was 44.7 pM (Fig. 8d)

Both the antibody-conjugated AuNP and AuNR SERS particles were also quantified using Raman spectroscopy. As done previously, SERS probes were diluted using a 1:2 serial dilution across the wells of the immunoassay plate. Raman spectra were acquired for each individual well in the plate, and a similar correlation between SERS probe concentration and light scattering signal was observed. Fig. 8a and Fig. 8c show the Raman spectrum of the AuNP and AuNR SERS probes, respectively. Both Raman spectra show characteristic peaks corresponding to DTTC iodide and to the polystyrene plate. In both cases, the spectral area of the DTTC iodide peak is correlated to the concentration of SERS probe, while the polystyrene peak intensity remains constant. A standard curve and sensitivity plot illustrating the correlation for both AuNP and AuNR SERS probes is shown in Fig. 8b and Fig. 8d, respectively. For both SERS platforms, the standard curve was fit using five-parameter logistic regression. The LLOD for each assay was 76.1 pM and 44.7 pM for AuNP and AuNR SERS probes, respectively.

Previous studies have suggested that to produce strong Raman enhancement, the nanoparticle size, shape, and composition should be chosen so that the particle LSPR peak is positioned near the excitation wavelength [64, 65]. To increase the sensitivity, strongly enhancing AuNRs where chosen for Raman reporter binding. Because the 779nm LSPR peak of the AuNR SERS probes was closer to the Raman system’s excitation wavelength, an increase in the SERS response was observed. The lower than expected optical absorbance and SERS response of AuNR particles may be due to particle aggregation as suggested by the wide LSPR peaks and lower intensities seen in optical absorbance spectroscopy (Fig. 7c). These results indicate that developing a fabrication schema for better control of the aggregation process may result in greater SERS and optical absorbance enhancement for this assay. Despite substrate interference and nanoparticle aggregation, both AuNP and AuNR assays had nanoparticle labeling concentrations that fell within the typical dynamic range for direct cell labeling (See Table 1).

Continued research on the optimal nanoparticle and Raman reporter combination for large Raman scattering enhancement on a polystyrene substrate may result in even greater enhancement and higher assay sensitivity. A promising application that may result in increased SERS assay sensitivity is the development of precisely engineered nanoparticle junctions (or hot spots) for much larger Raman enhancement. Electromagnetic hot spots between nanoparticle aggregates and the tips of nanostructures can result in signal enhancement of up to 10^8^ - 10^12^ times [27, 66, 67] and have been used for single molecule detection [68–74]. To promote assay reproducibility, hot spot nanoparticle aggregates must be precisely fabricated and characterized. Further work utilizing precisely engineered hot spot nanoparticle clusters may open the doors for a sensitive, multiplex SERS assay on a polystyrene substrate.

## Conclusion

This research article has described a step-by-step process to fabricate SERS probe nanoparticles using spherical gold nanoparticles and gold nanorods. The optical properties of both SERS probe platforms were tested in a light scattering immunoassay. This method for SERS probe fabrication and testing promoted reproducibility and consistency in the probe's optical response and allowed us to fabricate SERS probes with a signal that could be detected on a polystyrene substrate. The optical signal of the probes was detected using a dual-modality approach where both elastic and inelastic scattering were detected using optical absorbance spectroscopy and Raman spectroscopy, respectively. Despite substrate interference, both spherical gold nanoparticles and gold nanorods had assay detection limits in the low pM range. These results suggest that SERS-based cell labeling applications could be conducted on a polystyrene substrate. This SERS immunoassay method has potential for high-throughput, multiplex biomarker analysis. In the future, these probes may be used to detect multiple biomarkers in a clinical setting. If pursued, this method could enable the simultaneous, high-throughput detection of biomarkers specific to hematological malignancies or other complex diseases that require the detection of multiple biomarkers for successful diagnosis and treatment.

## Methods

### Design of a Raman microscope system

To determine the optical response of the SERS probes, a custom Raman microscope system was constructed. The system was retrofitted to a Nikon TE-2000-S inverted microscope base. A 785nm single-mode laser from Innovative Photonics Solutions (Monmouth, NJ) was coupled to a fiber optic cable with a numerical aperture of 0.39 and a core diameter of 400μm (Thorlabs Inc, Newton, NJ). The end of the fiber was attached to a laser entry port on the back of the microscope base to illuminate the sample on the microscope stage. Laser light was focused onto the sample and backscattered light was collected through the objective lens. A longpass filter removed elastic scattering and the Raman scattered light, which was passed to the spectrometer and dispersed using an IsoPlane 160 spectrometer (Princeton Instruments, Trenton, NJ) with a 1200 g/mm grating. The dispersed light was imaged with a Princeton Instruments Pixis 400 detector. All spectra were processed using LightField (Princeton Instruments) and Spekwin32 [75] spectral software. During Raman spectrum acquisition, 40mW of laser power was focused on the sample and Raman spectra were acquired using a 10-second acquisition time.

### Design of SERS probes and Raman reporter selection

60nm Citrate-capped gold nanoparticles were purchased from Ted Pella, Inc., Redding, CA. Citrate-capped gold nanorods were purchased from Nanocomposix, San Diego, CA. Raman reporter molecules including 4-aminothiolphenol (4-ATP), 4-mercaptobenzoic acid (4-MBA), crystal violet, malachite green, and 3,3′- diethylthiatricarbocyanine (DTTC) iodide were purchased from Sigma Aldrich, St. Louis, MO.

To produce SERS probes, 60nm citrate-capped gold nanoparticles (2.6x10^10^ nanoparticles per milliliter) were concentrated and added to a glass vial. The solution was rapidly stirred with a magnetic stir bar and the reporter molecules were added dropwise to the nanoparticles at a 1:6 volumetric ratio as used in prior work [5]. The optimal Raman reporter molecule was determined by surveying the optical response of several Raman reporter molecules including 4-ATP, 4-MBA, crystal violet, malachite green, and DTTC iodide. Each reporter colloid solution was incubated for 1 hour to promote surface binding.

### Antibody conjugation and particle functionalization

To develop antibody-conjugated SERS probes, cross-absorbed human and mouse IgG antibody-antigen pairs were used (Thermofisher, Rockford, IL). Antibodies were conjugated to the SERS probes using orthopyridyldisulfide-polyethylene glycol-succinimidyl valerate (OPSS-PEG-SVA, Laysan Bioscience). Prior to conjugation, the antibodies were transferred to a buffer solution of 100mM sodium bicarbonate using ZebaSpin™ desalting columns with a 10kDa molecular weight cutoff (Thermofisher). After buffer exchange, 5000 molecular weight OPSS-PEG-SVA was added to the protein solution. The reaction proceeded at room temperature for 2 hours and then overnight at 4 °C. After conjugation, the PEGylated antibodies were quantified using SDS-PAGE.

Using a TGX Fast Cast™ gel casing kit (BioRad, Hercules, CA), a 7.5 % polyacrylamide gel was prepared. PEGylated proteins were mixed with non-reducing lithium dodecyl sulfate sample buffer (Thermofisher) and placed in a water bath at 100 °C for 4 minutes. After cooling, the PEGylated proteins were loaded into the gel and the gel was run for 75 minutes at 150V using tris-HEPES-SDS running buffer (Thermofisher). The gel was stained for protein using a Coomassie Blue protein stain and for PEG using a 5 % barium chloride solution followed by a 1N iodine/iodide solution as done by other researchers [76–80].

To fabricate the SERS probe nanoparticles, PEGylated antibodies were bound to the gold nanoparticles surface by gold-disulfide binding [53]. DTTC iodide was added dropwise to the nanoparticles at a range of 200 - 1500μM and was incubated with the particles for 1 hour. Following incubation, PEGylated antibodies were added dropwise to the nanoparticle solution at an antibody to nanoparticle ratio of 200:1. The colloid solution incubated for 1 hour to promote disulfide bonding to the particle surface. Finally, SH-PEG was added to the particles at a concentration of 10μM to block any unbound surface sites and to increase particle stability. The SH-PEG was incubated for 10 minutes after which the particles were centrifuged at 5000xg, the supernatant was removed, and the particles were suspended in PBS to maintain protein stability and structural properties. The antibody-conjugated SERS probes were stored at 4 °C until use in the immunoassay.

### Light scattering immunoassay development

A light scattering immunoassay was developed using high-bind polystyrene plates as a binding substrate. IgG isotype antibodies were added to the plate surface at a concentration of 50μg/ml. After 1-hour incubation, the plate was washed with tris-buffered saline with 0.05 % tween 20 (TBST). To prevent non-specific binding, AAA superblock (Scytek Laboratories, Logan, UT), was added to each well of the plate for an additional hour. After incubation, the blocker was removed from the wells and SERS probes were added to the immunoassay plate. The SERS probes were incubated in the wells of the immunoassay plate for 2.5 hours to ensure that complete antigen binding had occurred. The plate was washed 3 times with TBST to remove unbound SERS probe.

Both AuNP and AuNR SERS probes were used to determine the SERS assay sensitivity. AuNP and AuNR SERS probes were fabricated according to the protocol above, except that prior to Raman reporter labeling, the particles were centrifuged and concentrated. The molar concentration of particles added to each well was estimated based on the initial nanoparticle concentration and the optical density of the nanoparticle solution after fabrication, assuming a linear relationship between nanoparticle concentration and optical density [81]. Binding of the SERS probe to the immunoassay plate was determined using optical absorbance spectroscopy and Raman spectroscopy. With optical absorbance spectroscopy, the absorbance of each well was measured at the LSPR wavelength. With Raman spectroscopy, the Raman scattering spectra were measured and baseline corrected to remove fluorescence.

The corrected spectra were normalized based on the intensity of the major polystyrene peak at 1002 cm^−1^, and the spectral area of each Raman reporter peak was calculated. The spectral peak area was compared to the concentration of SERS probe particles and a standard curve for the assay was developed by first fitting the data to a 5-parameter logistic curve. LLOD values were computed as outlined in the Clinical and Laboratory Standard Institutes, EP17-A2 protocol [61, 62]. Briefly, the limit of blank (LOB) and LLOD values were computed using the following equations: $LOB = {\overline{x}}_{Blank}+1.645{\sigma}_{Blank}$, *LLOD* = *LOB* + 1.645*σ*_*LowRange*_ where ${\overline{x}}_{Blank}$ is the mean value of the blank, *σ*_*Blank*_ is the standard deviation of the blank, and *σ*_*LowRange*_ is the standard deviation of lowest concentration sample present in the standard curve.

## Additional files

Additional file 1: Figure S1. Design of a custom Raman microscope system. The optical response of the SERS probes was detected using a custom Raman microscope system, which was constructed with an inverted microscope base, Figure S1A. With its inverted base and long working distance objectives lenses, the system was built to analyze SERS probes on a microplate substrate. The custom Raman system had a spectral resolution of 1.6 cm^−1^, a laser wavelength of 785nm, and a maximum power of 40mW. The Raman system captured spectral images using a 1340x400 pixel CCD detector. A Raman spectral image was obtained (Figure S1B, background image) and was averaged across each column to produce a Raman spectrum (Figure S1B, red overlay). The custom Raman system was used to acquire the Raman spectra of SERS probes in the development of light scattering immunoassays. (PDF 1673 kb) Additional file 2: Figure S2. SERS enhancement of different Raman reporters on spherical gold nanoparticles. Raman spectra were acquired for five different Raman reporter molecules; DTTC iodide, Crystal Violet, Malachite Green, 4-ATP, and 4-MBA. Sixty nanometer spherical gold nanoparticles were labeled with each reporter at a concentration of 400nM. After labeling, the Raman spectrum of each nanoparticle solution was acquired on our custom Raman microscope system. The intensity of the major peak in each spectrum was determined and compared. The Raman intensity of DTTC iodide is much greater than for other reporter molecules. (PDF 103 kb) Additional file 3: Figure S3. OPSS-PEG-SVA conjugation and hydrolysis. Activated OPSS-PEG-SVA can undergo two separate and competing reactions, hydrolysis and conjugation. To promote conjugation, the concentration of the protein solution was maintained at greater than 1mg/ml during conjugation and the NHS ester molecule was added to the protein solution immediately upon suspension. In addition, the NHS ester reagent was stored at −20 °C in a desiccator and under nitrogen to avoid possible hydrolysis due to ambient moisture. The hydrolysis reaction will release a NHS ester-leaving group with a maximum absorbance at 260nm. The quality of the stored NHS ester reagent was estimated by measuring the absorbance of a freshly prepared solution at 260nm and comparing that value to the initial absorbance from a newly opened vial of the reagent. (PDF 114 kb) Additional file 4: Figure S4. SERS probe stability and optical response. The stability of the SERS probes was determined by measuring the optical response of the probes before and after the addition of the SH-PEG. SERS probes fabricated using the synthesis methods described previously were stable for at least 1 month when stored at 4 °C. When SH-PEG was added to the gold nanoparticle solution, it did not significantly displace the Raman reporter. In addition, the aggregation state, optical response, and protein function were not adversely affected by centrifugation. (PDF 163 kb)

## Abbreviations

4-ATP: 4-Aminothiolphenol
4-MBA: 4-Mercaptobenzoic Acid
ELISA: Enzyme-Linked Immunosorbent Assay
DTTC: 3,3′- Diethylthiatricarbocyanine
LSPR: Localized Surface Plasmon Resonance
LLOD: Lower Limit of Detection
OPSS-PEG-SVA: orthopyridyldisulfide-polyethylene glycol-succinimidyl valerate
SDS-PAGE: Sodium Dodecyl Sulfate - Polyacrylamide Gel Electrophoresis
SERS: Surface-Enhanced Raman Spectroscopy
SH-PEG: Thiol-Polyethylene Glycol
