# Supplementary figures and images for: Rational design of Raman-labeled nanoparticles for a dual-modality, light scattering immunoassay on a polystyrene substrate
Source: J Biol Eng. 2016 Jan 7;10:2. doi: 10.1186/s13036-015-0023-y (PMC4705623; doi:10.1186/s13036-015-0023-y)

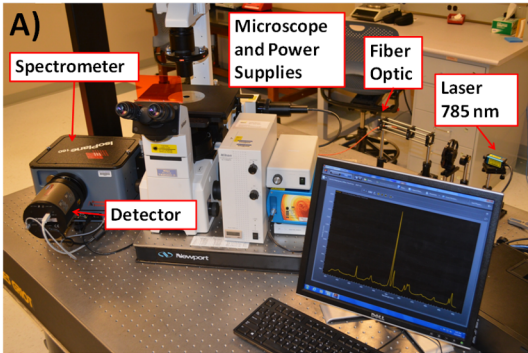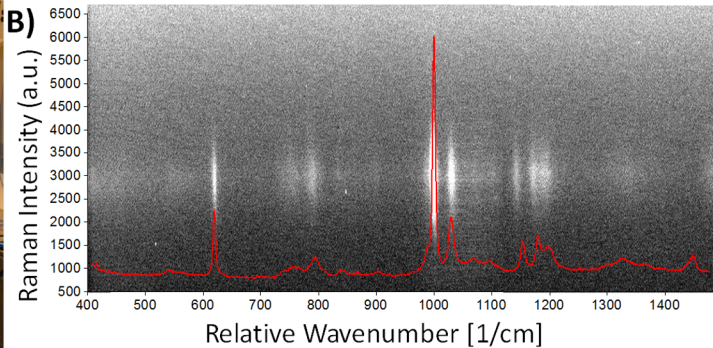

Supplement: Additional file 1: Figure S1. — Design of a custom Raman microscope system. The optical response of the SERS probes was detected using a custom Raman microscope system, which was constructed with an inverted microscope base, Figure S1A. With its inverted base and long working distance objectives lenses, the system was built to analyze SERS probes on a microplate substrate. The custom Raman system had a spectral resolution of 1.6 cm−1, a laser wavelength of 785nm, and a maximum power of 40mW. The Raman system captured spectral images using a 1340x400 pixel CCD detector. A Raman spectral image was obtained (Figure S1B, background image) and was averaged across each column to produce a Raman spectrum (Figure S1B, red overlay). The custom Raman system was used to acquire the Raman spectra of SERS probes in the development of light scattering immunoassays. (PDF 1673 kb) [file 13036_2015_23_MOESM1_ESM.pdf]

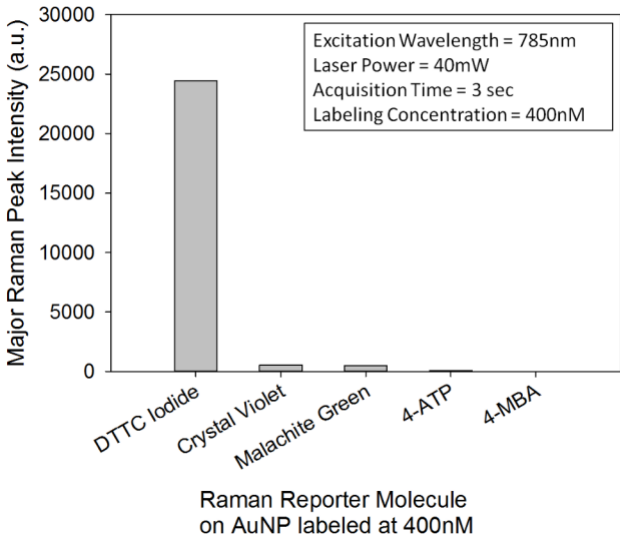

Supplement: Additional file 2: Figure S2. — SERS enhancement of different Raman reporters on spherical gold nanoparticles. Raman spectra were acquired for five different Raman reporter molecules; DTTC iodide, Crystal Violet, Malachite Green, 4-ATP, and 4-MBA. Sixty nanometer spherical gold nanoparticles were labeled with each reporter at a concentration of 400nM. After labeling, the Raman spectrum of each nanoparticle solution was acquired on our custom Raman microscope system. The intensity of the major peak in each spectrum was determined and compared. The Raman intensity of DTTC iodide is much greater than for other reporter molecules. (PDF 103 kb) [file 13036_2015_23_MOESM2_ESM.pdf]

# Conjugation

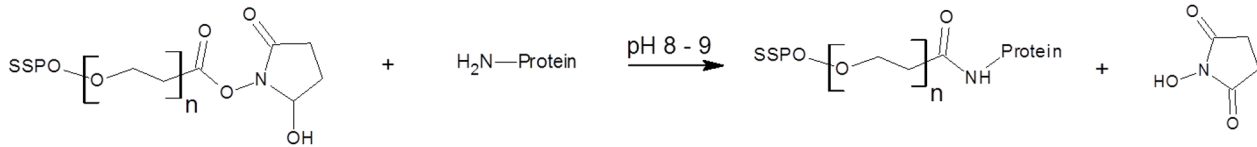

# Hydrolysis

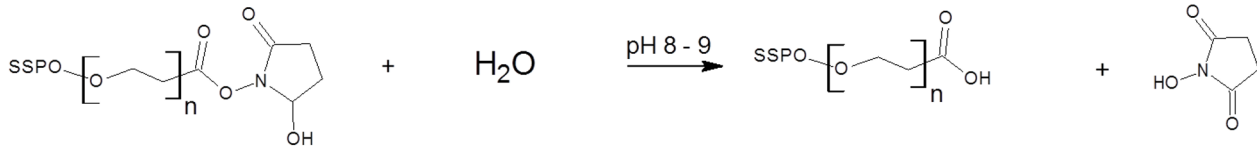

Supplement: Additional file 3: Figure S3. — OPSS-PEG-SVA conjugation and hydrolysis. Activated OPSS-PEG-SVA can undergo two separate and competing reactions, hydrolysis and conjugation. To promote conjugation, the concentration of the protein solution was maintained at greater than 1mg/ml during conjugation and the NHS ester molecule was added to the protein solution immediately upon suspension. In addition, the NHS ester reagent was stored at −20 °C in a desiccator and under nitrogen to avoid possible hydrolysis due to ambient moisture. The hydrolysis reaction will release a NHS ester-leaving group with a maximum absorbance at 260nm. The quality of the stored NHS ester reagent was estimated by measuring the absorbance of a freshly prepared solution at 260nm and comparing that value to the initial absorbance from a newly opened vial of the reagent. (PDF 114 kb) [file 13036_2015_23_MOESM3_ESM.pdf]

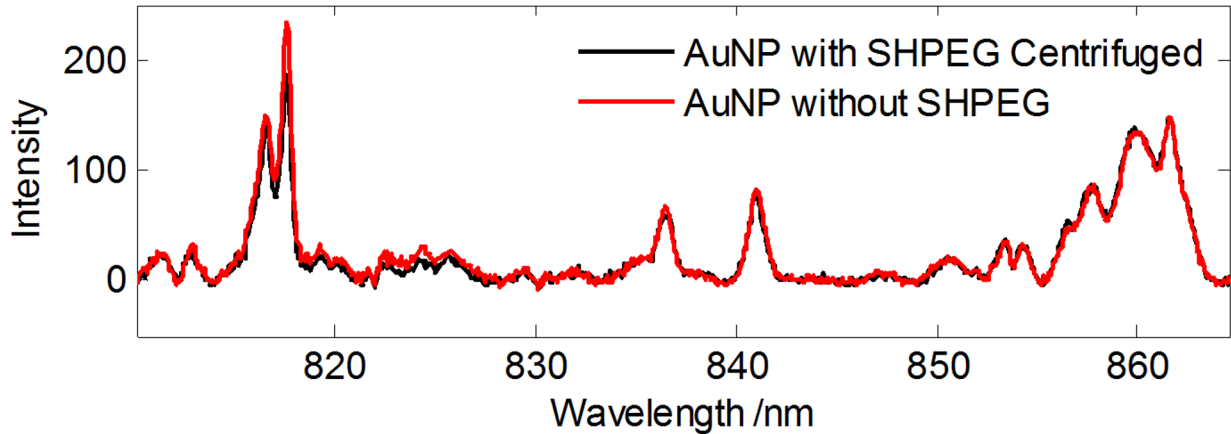

Supplement: Additional file 4: Figure S4. — SERS probe stability and optical response. The stability of the SERS probes was determined by measuring the optical response of the probes before and after the addition of the SH-PEG. SERS probes fabricated using the synthesis methods described previously were stable for at least 1 month when stored at 4 °C. When SH-PEG was added to the gold nanoparticle solution, it did not significantly displace the Raman reporter. In addition, the aggregation state, optical response, and protein function were not adversely affected by centrifugation. (PDF 163 kb) [file 13036_2015_23_MOESM4_ESM.pdf]
